# Incorporation of copaiba and oregano essential oils on the shelf life of fresh ground beef patties under display: Evaluation of their impact on quality parameters and sensory attributes

**DOI:** 10.1371/journal.pone.0272852

**Published:** 2022-08-10

**Authors:** Silvana Mari Belloli Leite, Esther Morais da Silva Assunção, Anandra Vitória das Neves Gurgel Alves, Edymeiko de Souza Maciel, Laura Adriane de Moraes Pinto, Isabelle Naemi Kaneko, Ana Guerrero, Ana Paula Folmer Correa, Jovanir Inês Müller Fernandes, Nívia Pires Lopes, Marcos José Salgado Vital, Jéssica de Oliveira Monteschio

**Affiliations:** 1 Department of Animal Science, Federal University of Roraima, Boa Vista, Roraima, Brazil; 2 Department of Animal Science, Federal University of Paraná, Palotina, Paraná, Brazil; 3 Department of Animal Science, Federal University of Rondônia Foundation, Rondônia, Brazil; 4 Facultad de Veterinaria, Departamento Producción y Sanidad Animal, Universidad Cardenal Herrera-CEU, CEU Universities, Alfara del Patriarca, Valencia, España, Spain; 5 Biodiversity Study Center, Federal University of Roraima, Boa Vista, Roraima, Brazil; University of Illinois, UNITED STATES

## Abstract

The preservative effect of the addition of different essential oils (copaiba and oregano) on meat quality parameters and sensorial acceptability was analyzed for fresh ground beef patties over 21 days of display. Five treatments were assessed: control (CON) without antioxidants; addition of the synthetic additive butylated hydroxytoluene (BHT); addition 0.05% of copaiba essential oil (CEO); 0.05% of oregano essential oil (OEO); or blend of 0.025% copaiba and 0.025% oregano essential oils (BEO). The lowest cooking losses and greatest tenderness (P <0.05) were reached with the blend (BEO). The inclusion of oregano essential oil presented a more intense chroma (P <0.05), with the best color retained during display. Oregano essential oil (OEO) and the blend (BEO) showed the highest antioxidant activity, reducing the lipid oxidation of beef patties during display (P < 0.05). Consumers preferred the odor of beef patties with essential oils (OEO and BEO) to the CON; however, the flavor from OEO had the lowest acceptability and the worst scores for overall acceptability (P < 0.05). Patties with the blend addition (BEO) were the best scored on overall acceptability assessments. In conclusion, the oregano and copaiba essential oils blend had a good preservative effect on fresh beef patties during display and increased sensory acceptability of the product, thus being a possible alternative for replacing synthetic compounds in processed foods.

## Introduction

Consumer demand for meat and meat products has increased quickly, mainly due to the high levels of nutrients in these products [1,2]. However, meat spoils quickly in external environments, and its deterioration is a process that involves the interactions of physical-chemical and microbial changes, with contamination by microorganisms and lipid oxidation during processing, storage, and display being the main causes of its reduced shelf life and consequently economic losses in the meat industry [3,4].

To meet industrial needs, processed products prove to be an attractive alternative, since the transformation of meat into processed products can generate nutritionally valuable by-products that can be used to add value, encouraging better use of that meat and increasing the industry profitability [2,5,6].

The addition of chemical preservatives can minimize or prevent lipid peroxidation, delaying the formation of toxic compounds and increasing the shelf life of foods [7]. Currently, most of the antioxidants added to meat products are chemically synthesized compounds, such as butylated hydroxyanisole (BHA) and butylated hydroxytoluene (BHT) [1]. The use of BHT as a food preservative has been limited in the food industries as antioxidants in food products, due to its association with a series of health hazards, such as the incidence of cancer, asthma, and behavioral disorders in children [8,9].

As an alternative to synthetic compounds, natural substances of plant origin such as essential oils have been widely applied in the meat industry as safe alternatives and approved for use in foods by the Food and Drug Administration [10] that exhibit antimicrobial and antioxidant properties [11–13].

Among the many oils approved for use, copaiba essential oil (*Copaifera* spp., CEO) is widely distributed in northern South America and produced in the Brazilian Amazon rainforest [14]. Its main bioactive components are phenolic compounds, such as sesquiterpenes and diterpenes [15]. The CEO has historically been used as a traditional medicine in the Amazon, there are indications that its intake in humans may be beneficial [16], and many studies have shown its use as a natural alternative to prevent diseases [17–19].

Oregano essential oil (*Origanum vulgare* L) is also one of the oils widely used as food preservatives, having different active phenolic compounds, such as thymol and carvacrol, providing good antioxidant activity [20–22]. In addition, its attractive palatability allows it to be successfully incorporated into food formulations [12]. In vitro and in vivo studies have reported the positive effects of oregano and its bioactive components on human health to neutralize chronic infectious and degenerative diseases [23,24].

This study aimed to evaluate the potential of the essential oils from copaiba and oregano as a natural antioxidants on beef patties over 21 days of display, assessing their antioxidant potential in preventing or delaying lipid oxidation and evaluating their influence on diverse meat quality parameters of the product along with its shelf life, as well as testing their sensory acceptance by consumers.

## Material and methods

### Preparation of beef patties

Meat to prepare patties came from the commercial cut striploin cap of four Nelore heifers (least than 24 months old, slaughter weight 356.6 ± 32.6 kg.), purchased directly in the slaughterhouse 24 hours after animals’ slaughter. Muscles were refrigerated and transported to the laboratory where the excess fat was removed. The beef was minced (6 mm, Mixer Philips Walita Viva Collection RI1366, Brazil) mixed, and compressed manually with 10 g of NaCl per kg of meat. Patties (40 g each) were produced in molds 10 cm in diameter and 1 cm in height, specific for the preparation of beef patties, and randomly distributed to the five different treatments tested with four replicates of four samples per each day of analyses per treatment.

Tested treatments were: a control without antioxidants (CON); beef patties containing synthetic additive butylated hydroxytoluene (BHT, 0.001%); beef patties with 0.05% of copaiba essential oil (CEO), beef patties with 0.05% of oregano essential oil (OEO) and beef patties with an essential oil blend: 0.025% copaiba essential oil + blend of 0.025% oregano essential oil (BEO). Afterward, the patties were placed on polystyrene trays coated with oxygen-permeable polyethylene film. The trays were exposed in a refrigerated showcase (4 ° C), under fluorescent light (380 lux, 12 h/day), simulating conditions typical of the Brazilian market.

The essential oils tested were obtained from Ferquima^®^ (Vargem Grande Paulista, São Paulo, Brazil), and the concentration used was based on previous research [5,12]. The main constituents of copaiba essential oil are sesquiterpene hydrocarbons (88.1%) and β-caryophyllene (51.1%) [5], and for the oregano essential oil, carvacrol (68.26%) and gamma-terpinene (8.01%) [25]. For BHT treatment, 10 mg/kg of butylated hydroxytoluene was added to beef patties according to de Carvalho et al. 2019 [26].

The samples for lipid oxidation (TBARS), antioxidant capacity (ABTS, DPPH, and TPC), pH, color properties, cooking losses, and texture were evaluated at (1, 7, 14, and 21 days of display. The chemical composition of the beef patties was performed only on the first day of display (production day) to define the basic meat composition.

### Characterization of beef patties (chemical composition)

The moisture, protein, lipid, and ash were measured in raw beef patties, according to the methods established by the Association of Official Analytical Chemists [27]. Crude protein was obtained by the semi-micro Kjeldahl method following three distinct steps: digestion, distillation, and titration [28].

### Antioxidant activity

Burger bioactive compounds extract. Beef patties extracts (1:1 w/v with methanol) were obtained using an ultra-Turrax homogenizer, followed by centrifugation and filtration. The extracts were analyzed for antioxidant activity by measuring the 2,2’-azino-bis (3-ethylbenzothiazoline-6-sulfonic) (ABTS) radical scavenging activities, and the total phenolic content (TPC) according to Monteschio et al. 2021 [5].

ABTS radical scavenging assay and Folin–Ciocalteu for total phenolic content (TPC). Beef samples were mixed with methanol (1:1 w/v with methanol) to obtain beef patties extracts for antioxidant analysis. Five grams of hamburger samples were ground with methanol using an Ultra-Turrax homogenizer, followed by centrifugation and filtration (4,000 rpm for 15 minutes). The extracts were analyzed for antioxidant activity by measuring the 2,2’-azino-bis(3-ethylbenzothiazoline- 6-sulfonic) (ABTS) radical scavenging activities, as well as the total phenolic content (TPC).

For the ABTS solution, 7mM ABTS (5 mL) was mixed with 140 mM potassium persulfate (88 μL), and the mixture was incubated in the dark at 25 °C for 16 h. The ABTS-activated radical was diluted with ethanol to an absorbance of 0.70 ± 0.02 at 734 nm. The radical scavenging activity (%) was also measured at 734 nm.

Beef patties extract (40 μL) was mixed with ABTS+• solution (1960 μL), and the absorbance was recorded after 6 min. The ABTS radical scavenging activity (%) was calculated as 1- (Asample t0/Asample t) × 100, where Asample t0 is the absorbance of the sample at time zero and A sample t is the absorbance of the sample at 6 min.

For TPC, an aliquot of each extract (125 mL) was mixed with 125 mL of Folin–Ciocalteu reagent (1:1 v/v deionized water) and 2250 mL Na_2_CO_3_ (28 g/L). The solutions were then incubated in the dark at 25 °C for 30 min, and the absorbance was measured at 725 nm using a spectrophotometer (Evolution 201 UV–visible spectrophotometer, Thermo Scientific), and the results were expressed as milligrams of gallic acid equivalents (GAE) per gram of sample. The ABTS radical scavenging activity (%) and TPC were calculated according to Monteschio et al. 2021 [5].

### Lipid oxidation

The malondialdehyde (MDA) content in beef patties was measured by the thiobarbituric acid-reactive substances (TBARS) assay with modifications [29]. The sample (5 g) was mixed with 15 mL of TCA solution (7.5% trichloroacetic acid, 0.1% gallic acid, and 0.1% EDTA), homogenized using an Ultra-Turrax, and then centrifuged for 15 min. The supernatant was filtered and mixed with TBA solution (1% thiobarbituric acid, 15% trichloroacetic acid, and 562.5 μM HCl). The mixture was boiled (100 ºC) for 15 min, cooled, and the absorbance at 535 nm was compared with an MDA standard. Results were expressed as mg MDA/kg of meat.

### Instrumental meat color

The CIELab color parameters were recorded by using a Minolta CR-400 chroma meter (Japan) under D65 illumination, aperture of 8 mm, and a closed cone set on the L*a*b* system, with a 10° view angle, obtaining lightness (L*), redness (a*), and yellowness (b*), Chroma (C*), and hue (*h*°) were calculated as follows:

$$C^{*}=(a^{*2}+b^{*2})$$

$$h{}^{\circ}=\left[arctan\left(b^{*}a^{*}\right)\right]$$

### pH measurements

The pH was measured using a pH meter with a Testo 205/206 penetration probe. The pH meter was calibrated at 20 °C using standard pH 4.0 and 7.0 buffers before use.

### Cooking losses

Beef patties were weighed and wrapped in aluminum foil. Each sample was cooked in a pre-heated grill (Grill Mondial Premium Max Grill G-07, Brazil) at 200 °C until the internal temperature reached 72 °C, which was monitored using an Incoterm internal thermocouple (145 mm; Incoterm LTDA, Brazil). The beef patties were removed from the heat and left at ambient temperature to cool to 25 °C. Each burger was weighed and the cooking losses were calculated each day as a percentage relative to the initial weight.

### Texture measurement

After recording the cooked weight, beef patties were cut into rectangular pieces of 1 cm^2^, and cross-section and texture were analyzed using a texture analyzer installed with a Warner–Bratzler blade and set with a 50-kg load cell and an operating speed of 2 mm/s, registering the maximum shear force (kg).

### Consumer acceptability

The sensory analysis study was approved by the Research Ethics Committee involving human beings at the Federal University of Roraima (protocol numbers: 23282819.9.0000.5302), Boa Vista, State of Roraima, Brazil, S1 File.

Before tasting each sample, each participant completed a written consent form, S1 File, containing all information about the experiment, and all questions regarding the procedures that would be performed in the research, and through this information, they could decide whether they would be volunteers for the study. The terms also exempt the participant from any liability concerning their health, transferring all responsibility to researchers.

In a room adequate to perform sensory analyses (individual cabins and without interference from external factors), 124 consumers participated in the sensory test; they were divided into 13 sessions with 10 participants per session, some consumers did not complete the test and were removed from the study. Consumers were randomly selected with quotas of gender (42% men and 58% women) and age (from 18 to 70 years) according to the Brazilian national profile.

Each consumer assessed five different samples (one from each treatment evaluated), after 1 day of display. A randomized design was used to serve the meat, avoiding carry-over and order effects. Beef patties were individually packed with aluminum foil and cooked on a pre-heated grill at 200 °C until the internal temperature reached 72 °C. Each beef patty was cut into four triangles and kept warm (50 °C) until consumer evaluation (around 10 min after cooking). Consumers scored acceptability of the following attributes of patties: odor, tenderness, flavor, and overall acceptability. Ratings were scored using a structured hedonic nine-point scale (1 = dislike extremely; 9 = like extremely), without the middle level.

### Microstructure

The microstructure analysis was performed according to Vital et al. 2016 [22] with modifications using a scanning electron microscope (SEM), at an acceleration voltage of 10 to 15 kV. Meat samples on day 1 of the display were dried in a circulating oven at 35 ° C ± 1 ° C for 72 hours, which were fixed and placed inside the chamber of the critical point drying instrument and dried using between one and eight cycles, to preserve microstructurally the samples. The samples were mounted in aluminum stubs and coated with a gold layer to observe the surface of the beef patties.

### Statistical analyses

All measurements were carried out in triplicate and data were analyzed as a factorial experiment in a completely randomized design using SPSS (version 27.0) (IBM SPSS Statistics, SPSS Inc., Chicago, IL, USA) for Windows. The factors included were five treatments (CON, BHT, CEO, OEO, and BEO) and four display times (1, 7, 14, and 21 days). A general linear model ANOVA assessed treatments and displays, which were considered fixed factors, and their interactions were also considered. For the consumer, acceptability treatment was the only fixed effect evaluated and the consumer was considered a random effect, and session a blocking effect.

When differences between means were statistically significant, Tukey’s test was performed, with statistical significance set at (P < 0.050).

## Results

### Physic-chemical analyses of beef patties

The composition of beef patties (Table 1) was similar for all treatments (P> 0.05), and it followed the Technical Regulation on Hamburger Identity and Quality [30], with lipid levels below 23% and the at least 15% protein.

**Table 1:** Chemical composition of beef patties treated with BHT, copaiba and oregano essential oil or blend of essential oils.

	CON^1^	BHT^2^	CEO^3^	OEO^4^	BEO^5^	SEM^6^	*P<*
**Moisture (%)**	60.92	60.70	60.83	60.86	60.80	0.081	0.960
**Ash (%)**	1.50	1.51	1.54	1.52	1.53	0.012	0.869
**Protein (%)**	21.46	21.56	21.60	21.64	21.76	0.112	0.960
**Fat (%)**	12.31	12.19	12.54	12.95	12.32	0.118	0.277

^1^CON–beef patties, without antioxidants;

^2^BHT—beef patties containing hydroxytolueneobutylated;

^3^CEO-0.05% beef patties with 0.05% of Copaiba essential oil;

^4^OEO- beef patties with 0.05% of Oregano essential oil;

^5^BEO—blend of essential oil (0.025% of Copaiba essential oil + 0.025% of Oregano essential oil).

^6^SEM: Standard error of means

### Antioxidant activity

The antioxidant capacity (ABTS and TPC) was affected by the treatments and display (P <0.05, Table 2). The ABTS antioxidant activity for beef patties was higher for OEO and BEO treatments, intermediate for BHT and CEO, and lower for CON.

**Table 2:** Evolution of lipid oxidation (TBARs), antioxidant activity ABTS, and total phenolic compounds treated with BHT, copaiba and oregano essential oil or blend of essential oils during storage.

	Treatments					Storage (Days)					P < Value		
Item	CON^1^	BHT^2^	CEO^3^	OEO^4^	BEO^5^	1	7	14	21	SEM^6^	T^7^	D^8^	TxD^9^
**Abts**	28.15^c^	37.55^b^	39.20^b^	51.42^a^	52.77^a^	29.90^D^	33.79^C^	59.41^A^	44.18^B^	2.597	0.000	0.000	0.000
**Polyphenols**	30.76^c^	33.50^b^	32.84^bc^	44.83^a^	45.97^a^	27.23^D^	32.87^C^	47.22^A^	43.00^B^	1.686	0.000	0.000	0.000
**Tbars**	0.98^a^	0.72^b^	0,73^b^	0.56^c^	0.55^c^	0.20^A^	0.41^B^	1.03^C^	1.18^D^	0.059	0.000	0.000	0.000

Means of treatments with different lower-case letters in the same line are significantly different (P < 0.05). Means of display with different uppercase letters in the same line are significantly different (P < 0.05).

^1^CON–beef patties, without antioxidants;

^2^BHT—beef patties containing hydroxytolueneobutylated;

^3^CEO-0.05% beef patties with 0.05% of Copaiba essential oil;

^4^OEO- burger with 0.05% of Oregano essential oil;

^5^BEO—blend of essential oil (0.025% of Copaiba essential oil + 0.025% of Oregano essential oil).

^6^SEM: Standard error of means;

^7^T = P value effect of treatment;

^8^D = P value effect days of storage;

^9^TxD = P value interaction between treatments and days of storage.

**Fig 1:**
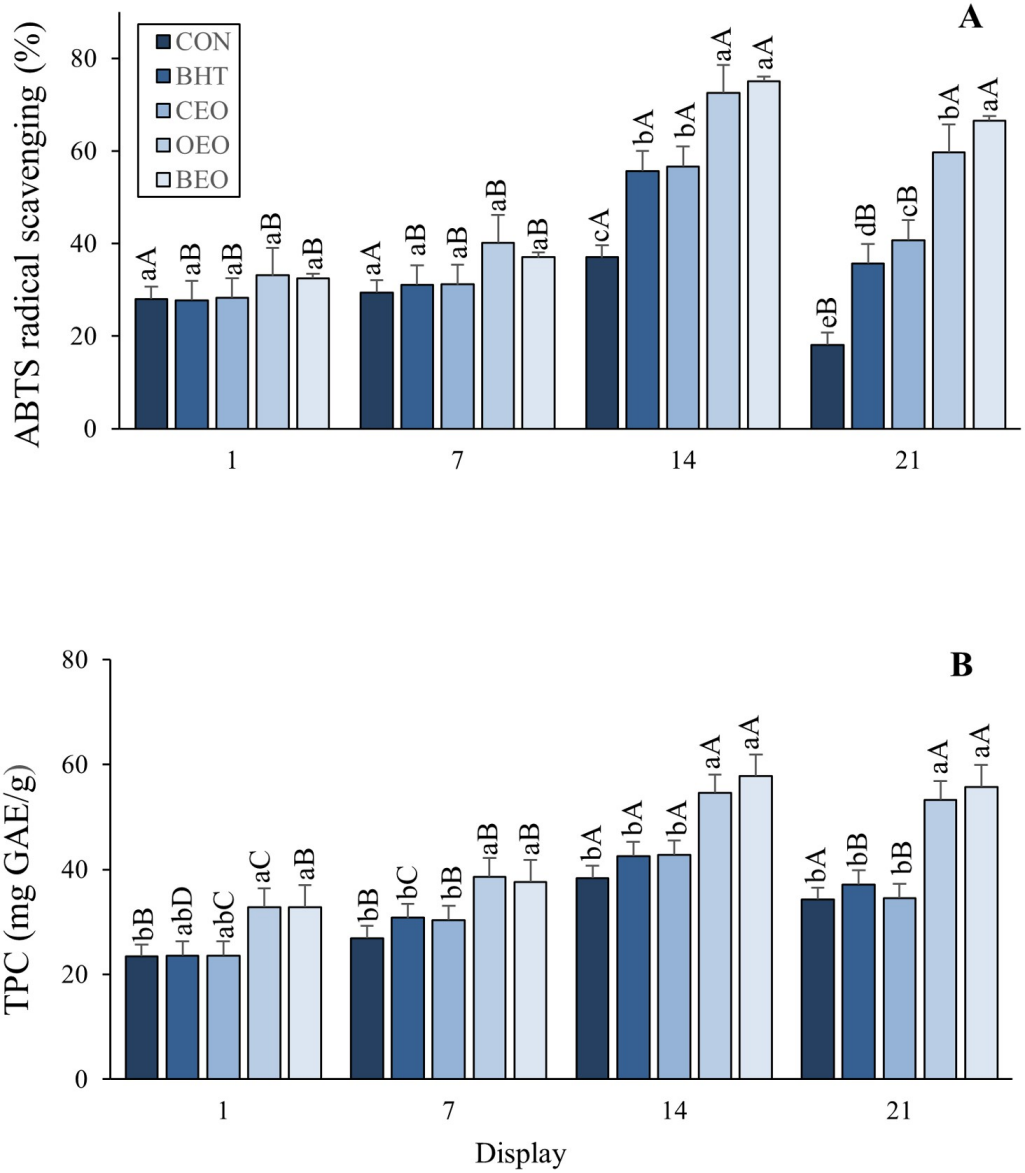
Evolution of antioxidant activity ABTS and total phenolic compounds in beef patties treated with BHT, copaiba and oregano essential oil or blend of essential oils during display.

There was a significant decrease (P <0.05) during display until day 7, with an increase on day 14 and a further decrease until the last day of display (day 21). An interaction between the effects (treatments and storage time) was observed, as is shown in Fig 1. On day 14 of display, the OEO and BEO treatments showed greater antioxidant activity than CON, BHT, and CEO intermediate activity. Greater antioxidant activity was observed for treatments with the addition of essential oils (CEO, OEO, and BEO), followed by BHT and CON after 21 days of cold display.

From the beginning of display (day 1) until day 14, the CON treatment showed greater antioxidant activity, decreasing on the last day of display (Day 21). BHT and CEO showed the same behavior of antioxidant capacity for display days, with higher values on day 14 between the other days (1, 7, and 21). Until day 7, the OEO and BEO treatments had lower antioxidant capacities and higher values on days 14 and 21 of the display.

In the TPC assay, activities similar to ABTS were observed for treatments and display days (P <0.05), showing greater antioxidant activity for OEO and BEO, lower CON, intermediate BHT, and the CEO not differing from CON and BHT. During display, the same behavior of ABTS was observed, showing a decrease in antioxidant activity until day 7, with an increase on day 14 and a further decrease until the last day of display (day 21). An interaction between the effects (treatments and display) was also observed for the CPT, as shown in Fig 1. Differences between treatments (P <0.05) were observed, with greater antioxidant activity found in OEO and BEO beef patties compared to the CON treatment and intermediate values for BHT and CEO on the first day of display. The OEO and BEO treatments showed greater antioxidant activity than the other treatments (CON, BHT, and CEO) on days 7, 14, and 21 of cold display. During storage, on days 1 and 7, the CON and BEO treatments showed similar activities, showing lower (P <0.05) antioxidant activities than on days 14 and 21 of display. On the fourteenth day of display, the BHT treatment showed the highest antioxidant TPC activity, followed by days 21, 7, and 1 of display. On day 14, the beef patties from the CEO treatment showed higher values by the TPC test compared to the first day and intermediate values on days 7 and 21 of display. On days 14 and 21 of display, the OEO showed greater antioxidant activity, followed by days 7 and 1 of the refrigerated display of beef patties.

Different lower-case letters indicate significant differences between treatments in the same day of display (P < 0.050). Different upper case letters indicate significant differences in the same treatment along display (P < 0.050). CON–beef patties, without antioxidants; BHT—beef patties containing hydroxytolueneobutylated; CEO-0.05% beef patties with 0.05% of Copaiba essential oil; OEO- burger with 0.05% of Oregano essential oil; BEO—blend of essential oil (0.025% of Copaiba essential oil + 0.025% of Oregano essential oil).

### Lipid oxidation

**Fig 2:**
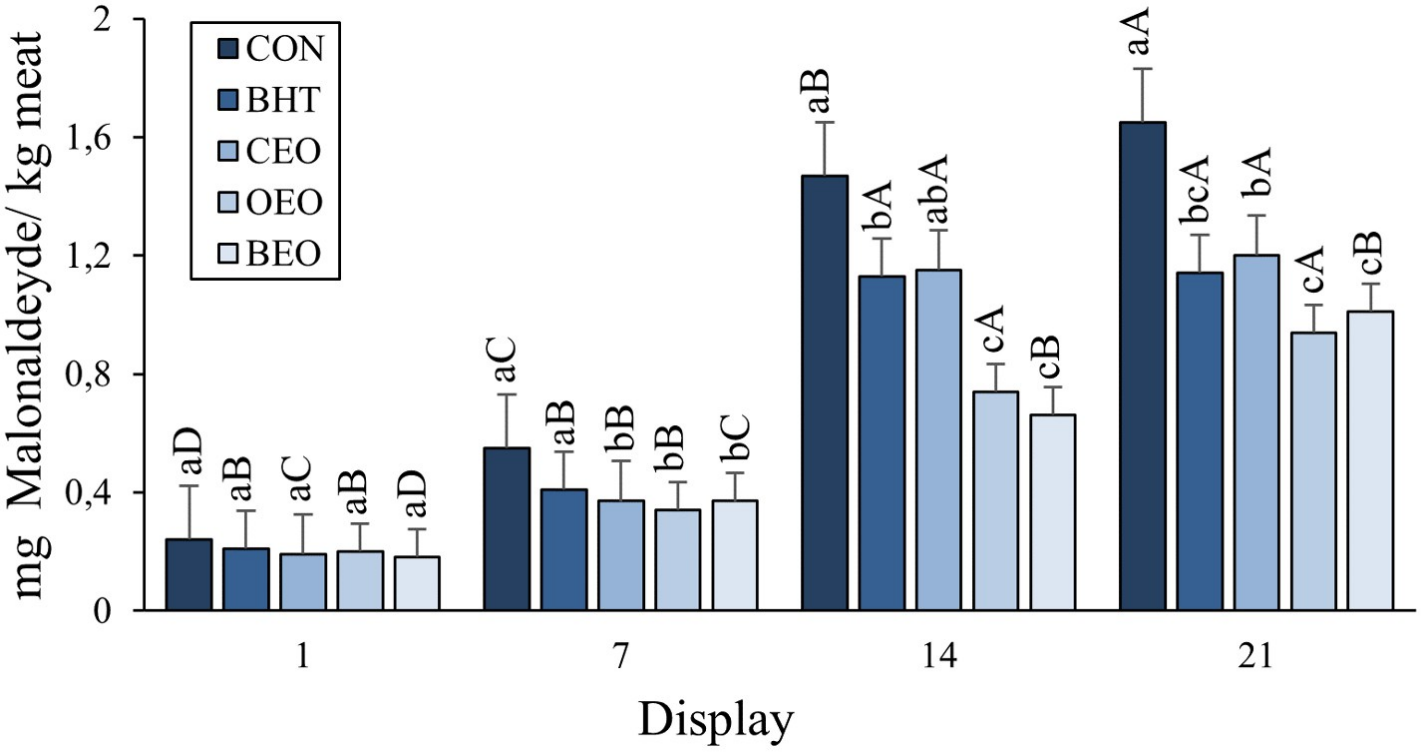
Evolution of lipid oxidation in beef patties treated with BHT, copaiba and oregano essential oil or blend of essential oils during display.

Lipid oxidation measured by MDA production was affected by treatments and display time (P <0.05). The CON showed a higher MDA value than OEO and BEO and intermediate values were found for the BHT and CEO treatments. In addition, the lipid oxidation of beef patties increased significantly (P <0.05) during the cold display. When the effects of both factors were assessed, an interaction was observed between them (Table 2), as shown in Fig 2.

Lipid oxidation was higher for the CON and BHT treatments compared to the other treatments on day 7 of display. The CON treatment showed a higher MDA value than the OEO and BEO, the BHT showed an intermediate value and the CEO treatment did not differ from the CON and BHT on day14 of display. On the last day of display (day 21), the CON presented a higher MDA value than the OEO and BEO, intermediate for the CEO and the BHT did not differ from the CEO, OEO, and BEO treatments.

Different lower-case letters indicate significant differences between treatments in the same day of display (P < 0.050). Different upper case letters indicate significant differences in the same treatment along display (P < 0.050). CON–beef patties, without antioxidants; BHT—beef patties containing hydroxytolueneobutylated; CEO-0.05% beef patties with 0.05% of Copaiba essential oil; OEO- burger with 0.05% of Oregano essential oil; BEO—blend of essential oil (0.025% of Copaiba essential oil + 0.025% of Oregano essential oil).

### Color parameters

The color values (L*, a*, b*, C*, and h°) associated with the treatments and display are shown in (Table 3). There were no differences (P > 0.05) between treatments for the values of L*, a*, b*, and hº.

**Table 3:** Evolution of CIE color parameters (L*, a*, b*, C* and H*) in beef patties treated with BHT, copaiba and oregano essential oil or blend of essential oils during storage.

	Treatments					Storage (Days)					P < Value		
Item	CON^1^	BHT^2^	CEO^3^	OEO^4^	BEO^5^	1	7	14	21	SEM^6^	T^7^	D^8^	TxD^9^
L*	40.56	37.31	39.10	40.76	40.48	40.99	37.88	39.63	40.03	0.730	0.558	0.451	0.334
a*	13.44	11.76	11.18	12.82	12.90	16.55A	12.04B	10.68BC	9.74C	0.407	0.136	0.000	0.667
B*	9.55ab	8.39b	9.23ab	12.18a	10.81ab	13.33A	9.20B	8.40B	8.79B	0.486	0.082	0.000	0.385
C*	16.59ab	14.58b	14.63b	18.12a	16.97ab	21.73A	15.28B	13.69B	13.19B	0.557	0.030	0.000	0.436
H*	36.64	35.64	39.72	41.38	39.75	37.12	37.68	38.26	41.90	0.947	0.358	0.305	0.524

Means of treatments with different lower-case letters in the same line are significantly different (P < 0.05). Means of display with different uppercase letters in the same line are significantly different (P < 0.05).

^1^CON–beef patties, without antioxidants;

^2^BHT—beef patties containing hydroxytolueneobutylated;

^3^CEO-0.05% beef patties with 0.05% of Copaiba essential oil;

^4^OEO- burger with 0.05% of Oregano essential oil;

^5^BEO—blend of essential oil (0.025% of Copaiba essential oil + 0.025% of Oregano essential oil).

^6^SEM: Standard error of means;

^7^T = P value effect of treatment;

^8^D = P value effect days of storage;

^9^TxD = P value interaction between treatments and days of storage.

Chroma (C*) showed a significant difference (P <0.05) between treatments, with a higher value for beef patties with the addition of essential oil of oregano, intermediate for the treatment BEO and control, and lower value for the treatment BHT and OEO.

Differences were observed for display days (P <0.05) for the values of a*, b*, and C*. Higher values of a* (redness) were observed at the beginning of display (day 1) and lower on the last day (day 21), intermediates found at days 7 and 14 of display, and day 14 did not differ from days 7 and 21 of display.

At the beginning of display (day 1), the values of b* (yellowness) were higher (P <0.05) and remained constant with the increase in display days. The same behavior was observed for the chroma values (C*) during the days of display of beef patties. No interactions were observed between treatments and display for color parameters.

### pH, cooking loss, and texture

The pH showed no differences (P >0.05) between treatments; however significant changes (P <0.05) occurred in display, with higher values ​observed at 21 days compared to shorter display periods (1 and 7 days) and intermediate values at 14 days of display (Table 4).

**Table 4:** Effect of BHT, copaiba and oregano essential oil or blend of essential oils on pH, cooking losses and shear force of beef patties during storage.

	Treatments					Storage (Days)					P < Value		
Item	CON^1^	BHT^2^	CEO^3^	OEO^4^	BEO^5^	1	7	14	21	SEM^6^	T^7^	D^8^	TxD^9^
**pH**	6.26	6.23	6.14	6.18	6.18	5.53C	5.59C	6.57B	7.09A	0.106	0.124	0.000	0.896
**Cooking loss,%**	24.39a	24.80a	24.24a	22.98a	20.97b	31.12A	29.12B	18.67C	14.99D	1.139	0.000	0.000	0.009
**Shear force, kg**	2.17a	1.97ab	1.90b	1.84b	1.57c	2.27A	2.23A	1.77B	1.29C	0.052	0.000	0.000	0.256

Means of treatments with different lower-case letters in the same line are significantly different (P < 0.05). Means of display with different uppercase letters in the same line are significantly different (P < 0.05).

^1^CON–beef patties, without antioxidants;

^2^BHT—beef patties containing hydroxytolueneobutylated;

^3^CEO-0.05% beef patties with 0.05% of Copaiba essential oil;

^4^OEO- burger with 0.05% of Oregano essential oil;

^5^BEO—blend of essential oil (0.025% of Copaiba essential oil + 0.025% of Oregano essential oil).

^6^SEM: Standard error of means;

^7^T = P value effect of treatment;

^8^D = P value effect days of storage;

^9^TxD = P value interaction between treatments and days of storage.

**Fig 3:**
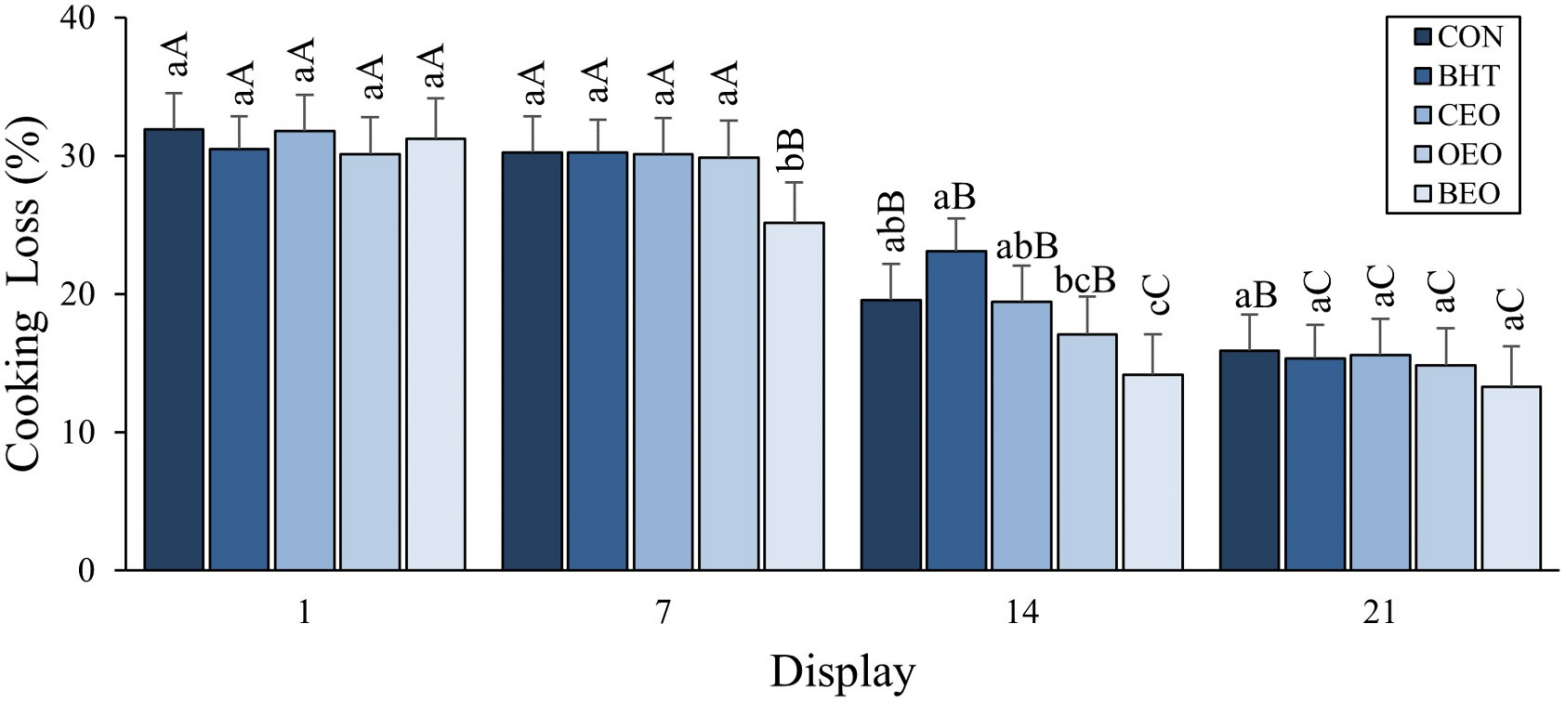
Evolution of cooking losses in beef patties treated with BHT, copaiba and oregano essential oil or blend of essential oils during display.

The treatments and days of display affected cooking losses (P <0.05). They were significantly lower at the end of display than on day 1, and the BEO treatment had the lowest cooking loss value among the other treatments (CON, BHT, CEO, and OEO). No interaction between treatments and display was observed for pH, while an interaction was observed for cooking loss, as shown in Fig 3.

Differences in the cooking loss were observed for the BEO treatment (P <0.05), which showed lower losses between the treatments evaluated at 7 days of display and for the BHT treatment at day 14 of display, which showed greater losses compared to the BEO, being that the CON and OEC treatment did not differ from BHT and the OEO and OEO treatment did not differ from CON, OEC, and BEO.

On display days, the differences found (P <0.05) showed the same behavior for the CON and OEO treatments with greater cooking losses with shorter display periods (days 1 and 7) compared to the other days. The BHT and CEO treatments showed greater losses on 1 and 7 days of display, were intermediate at 14 days, and lower at 21 days of display. The BEO treatment showed higher losses on the initial display day (day 1), was intermediate at 7 days, and had lower losses on the final display periods (days 14 and 21).

Regarding the tenderness of beef patties, differences (P <0.05) were observed between treatments and display periods. Softness was lower for the CON treatment than the BEO treatment, intermediate for the CEO and OEO treatments, and the BHT treatment did not differ from the CON, CEO, and OEO treatments. The shear force was greater until the seventh day of display, and from day14 onwards, there was a progressive reduction in values, indicating softer meat after a longer display (P <0.05). No interactions were observed between treatments and the display on the tenderness of beef patties (Table 4).

Different lower-case letters indicate significant differences between treatments in the same day of display (P < 0.050). Different upper case letters indicate significant differences in the same treatment along display (P < 0.050). CON–beef patties, without antioxidants; BHT—beef patties containing hydroxytolueneobutylated; CEO-0.05% beef patties with 0.05% of Copaiba essential oil; OEO- burger with 0.05% of Oregano essential oil; BEO—blend of essential oil (0.025% of Copaiba essential oil + 0.025% of Oregano essential oil).

### Consumer acceptability

For all parameters evaluated in sensory acceptability (odor, flavor, tenderness, and overall acceptability), differences (P <0.05) were observed between treatments (Table 5).

**Table 5:** Consumer acceptability of beef patties treated with BHT, copaiba and oregano essential oil or blend of essential oils (*n* = 124 consumers)^§^.

	CON^1^	BHT^2^	CEO^3^	OEO^4^	BEO^5^	SEM^6^	*P<*
**Odor**	6.60b	7.05ab	6.94ab	7.15a	7.25a	0.074	0.005
**Flavor**	6.73b	6.94ab	6.81b	5.99c	7.41a	0.078	0.000
**Tenderness**	6.65b	7.27a	7.26a	7.23a	7.43a	0.061	0.000
**Overall acceptability**	6.84b	6.77b	6.91b	6.19c	7.43a	0.074	0.000

^§^ Based on a 9-point scale (1: dislike extremely; 9: like extremely).

Means of treatments with different lower-case letters in the same line are significantly different (P < 0.05).

^1^CON–beef patties, without antioxidants;

^2^BHT—beef patties containing hydroxytolueneobutylated;

^3^CEO-0.05% beef patties with 0.05% of Copaiba essential oil;

^4^OEO- beef patties with 0.05% of Oregano essential oil;

^5^BEO—blend of essential oil (0.025% of Copaiba essential oil + 0.025% of Oregano essential oil).

^6^SEM: Standard error of means.

The odor obtained a higher score (P <0.05) for BEO and OEO compared to CON and intermediate values for the BHT and CEO treatments. For beef patties flavor acceptability, the highest scores (P <0.05) were found in the BEO treatment and lowest in the OEO treatment, being intermediate for the CON and CEO treatments, and the BHT treatment did not differ from the CON, CEO, and BEO treatments. The tenderness of beef patties from the CON treatment had the lowest score (P <0.05) significantly different from the other treatments (BHT, CEO, OEO, and BEO). The highest score for overall acceptability (P <0.05) was obtained for the BEO treatment, the lowest for the OEO treatment, and the intermediate scores were for the CON, BHT, and CEO treatments.

### Microstructure

The microstructure was examined to verify the structural properties of the processed food and the organization of the droplets of the essential oil present in the beef patties.

**Fig 4:**
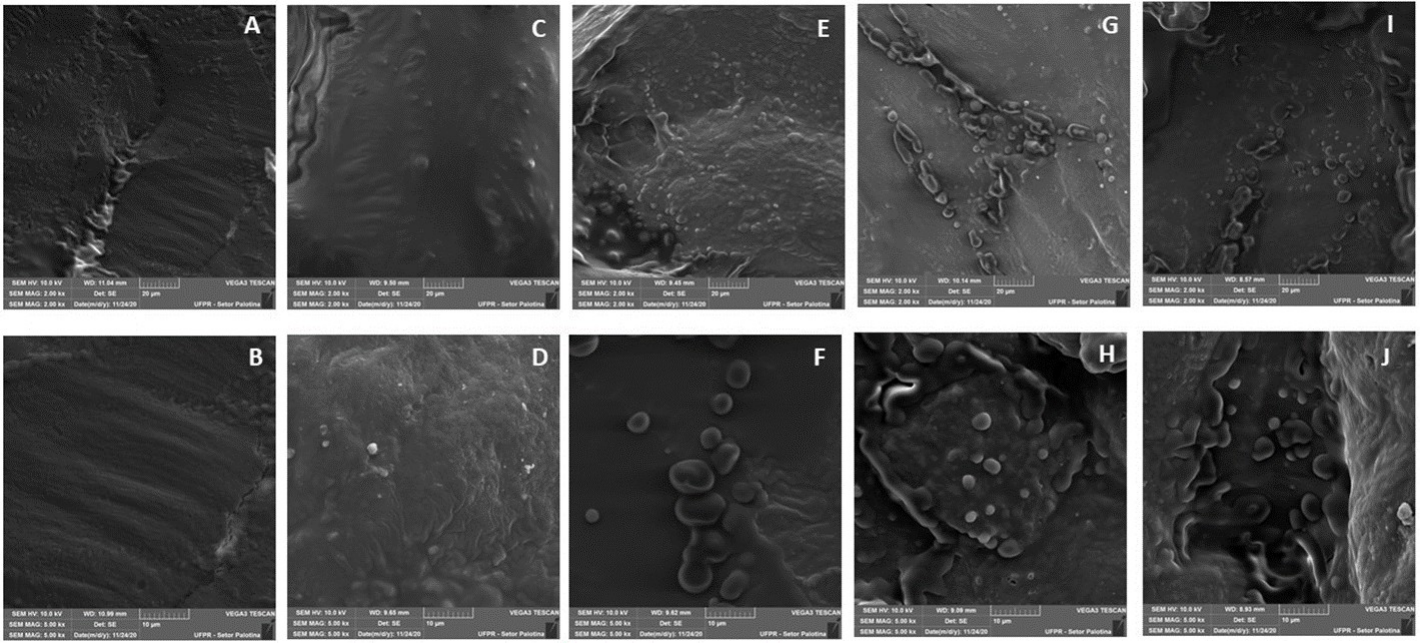
Scanning electron micrographs of the beef patties without oil essential e com BHT (A, B, C e D) and with essential oils (E an F—Copaiba), (G and H—Oregano) and (I and J—blend of essential oil). Magnification of 1200X, 1800X.

Microstructures of meat and essential oil are shown in (Fig 4). Beef patties without essential oil (Fig 4A and 4B) and with BHT were smooth and homogeneous (Fig 4C and 4D, beef patties with 1200 and 1800X magnification). In contrast, beef patties with copaiba essential oil, oregano, and the mixture of oils exhibited similar heterogeneous structures, with oil droplets dispersed throughout their structure (Fig 4E–4J, beef patties with 1200 and 1800X magnification).

## Discussion

### Physic-chemical analyses of beef patties

The percentage of fat in the patties of the current study is similar to those (which ranged from 11.84% to 13.05%) described by Borella et al. 2019 [31] for a mixed hamburger with the addition of rosemary (*Rosmarinus officinalis*) as an antioxidant.

Carvalho et al. 2019 [32] observed similar values of moisture (60.71% to 61.70%) and protein (19.71% to 19.74%) with the use of oregano essential oil as a natural antioxidant in meat hamburgers. Values higher than this study for ash (2.45% to 2.49%) were reported by Fachinello et al. 2018 [33] when evaluating the addition of freeze-dried green tea to hamburgers as a source of antioxidant during storage. The addition of additives, natural or synthetic, did not modify the basal chemical composition of the meat product and it was elaborated according to the standard product composition.

### Antioxidant activity

Phenolic compounds from plants are responsible for their antioxidant properties [34]; those and other kinds of compounds can donate a hydrogen atom to free radicals and thus delay or inhibit lipid oxidation, which is a major problem in food processing [35].

For the antioxidant activities evaluated by ABTS and TPC tests, beef patties containing OEO and BEO showed greater antioxidant activity compared to the CON treatment. The fact can be explained by the effectiveness of antioxidants, mainly natural antioxidants, and corroborate the work of other authors [12,26,36], who noticed that natural essential oils and extracts had more antioxidant activity than synthetic antioxidants, suggesting the possibility of using these extracts as substitutes for commercial additives.

In addition, the antioxidant capacity of oregano essential oil (OEO) and the mixture of essential oils (BEO) remained high until day 14 of display by the two methods of antioxidant capacity (DPPH and TPC), indicating that the polyphenolic compounds’ assets of these oils kept their activity throughout the display. The same increase in antioxidant capacity (DPPH) during display days was observed by Cantú-Valdéz et al. 2020 [37] when evaluating Mexican oregano essential oils as alternatives to butylated hydroxytoluene to improve the shelf life of ground beef.

### Evolution of lipid oxidation during storage

Changes in the oxidative state during display affect the shelf life of meat and its by-products [38], including lipid oxidation, is one of the main factors that limit the shelf life of muscle foods [39].

The use of essential oils (CEO, OEO, and BEO) was more effective than the CON and BHT treatments in delaying lipid oxidation after 7 days of display. This is due to the antioxidant activity of phytochemicals in the essential oil associated with the hydroxyl group attached to the aromatic ring, which is capable of donating hydrogen atoms with electrons and neutralizing free radicals [40,41], with the same observed for OEO and BEO at 14 days of display and OEO compared to CON at 21 days of display.

During the display period, TBARS values increased, which would be expected, as lipid oxidation is an autocatalytic reaction and, therefore, the oxidation rate increases as the reaction proceeds [39]. Despite the general increase in TBARS, the values on day 21 of the display were within the limit for the acceptability of oxidized beef and would be 2 mg MDA kg^−1^ according to Campo et al. 2006 [42], showing lower oxidations of the OEO and BEO treatments compared to CON, which may be related to phenolic compounds and antioxidant activity present in oregano essential oil [12] and copaiba essential oil [5].

Similar results were found by other authors when they incorporated sage in beef burgers during prolonged chilled storage [36], as well as chestnut extracts on the shelf-life of beef patties [43], basil (*Ocimum basilicum* L.) essential oil in beef burgers [44] and copaıba essential oil on the shelf life of sheep burgers [5]. The incorporation of the cited natural additives on burger elaboration improved lipid stability, delaying its oxidation.

### Color parameters

Redness a* is an important component in the visual appeal of meat by consumers and is related to the oxygenation of myoglobin, with high values of a* are associated with the presence of oxymyoglobin, while the reduction in a* values is related to the formation of metmyoglobin [4,45].

In the current experiment, it was expected that redness (a*) would be progressively reduced during display, as has been previously reported in other studies [1,46]. This discoloration of meat during cold display is caused by the loss of its ability to reduce metmyoglobin to oxymyoglobin, as well as lipid oxidation products and amine groups to react non-enzymatically in meat [47]. The same behavior was observed for the yellowing values (b*) that showed a decrease during storage. Regarding the display periods, the decrease in the intensity of red (a*) and yellow (b*) of hamburgers was observed in this study and was also reported by other authors. Teixeira et al. 2013 [48] observed a significant reduction in a* and b*, of hamburgers made with moringa leaves as a natural antioxidant. Fachinello et al. 2018 [33] also observed the same behavior with green tea as a source of antioxidants in frozen hamburgers.

The meat product with low chroma values is considered pale and clear [49], which may not be attractive to consumers at the time of purchase [22]. In this study, the OEO treatment had the highest C* value compared to BHT. De Lima et al. 2020 [50] also observed differences between treatments when evaluating the inclusion of essential oils in the substitution of synthetic antioxidants in Tambaqui fish meatballs with higher C* values with the addition of oregano essential oil. The same was observed by Vital et al. 2016 [22] who found higher C* values in meats coated with rosemary and oregano essential oil and reduced values during display days.

### pH, cooking loss, and texture

During display, the pH values increased, which reflects the degree of deterioration of the meat through the breakdown of proteins for the production of free amino acids leading to the formation of NH_3_ and amines, alkaline compounds [39,51], leading to a negative effect on product quality during display, mainly in terms of sensory characteristics such as odor, color, and texture [52]. Previously, similar findings in ground meat samples wrapped with the films containing *Zataria multiflora* essential oil have been reported by Amiri et al. 2019 [2]. Similar results are found in the literature [4,38].

The loss from cooking food is an important quality characteristic, associated with the meat yield at the moment of consumption, which can be influenced by the water retention capacity in the meat structures, providing better quality attributes to the food [50]. Beef patties from the BEO treatment showed lower cooking losses at 7 days of display compared to CON, and also at 14 days of display compared to BHT, which provides better texture and juiciness of the food [53], in addition to being desirable, since the presence of exudate is not attractive [54]. Thus, the mixture of essential oils showed protection to the meat against water losses.

The increase in cooking losses during display is one of the main undesirable changes in quality during shelf life, which was observed in this trial and results agree with other research results with essential oil blends on the meat of crossbred heifers [55] and essential oils in beef patties with copaiba oil [5].

The greater tenderness of the beef patties from the BEO treatment may be associated with water loss and oxidative processes during display days, and the presence of water in the product system provides a more succulent and softer product [56]. This fact can be confirmed by the BEO treatment having presented lower losses due to cooking and oxidation relative to the other treatments. Some authors evaluated the use of essential oils and extracts during display in meat products and found an increase in softness with the days of display [22,57].

### Consumer acceptability

The acceptability of food by consumers establishes the future direction of the market and influences the applications of new technologies in the food processing industry [37,58]. In the current study, the highest consumer scores for the odor were in beef patties from the OEO and BEO treatments compared to the CON treatment, and the lowest scores for flavor parameters and overall acceptability of OEO treatment compared to the BEO treatment. Consumer behavior can be associated with previous personal and culinary experiences consumers [59]. Oregano odor had a positive effect when samples were smelt; however, it was negative for the flavor (which includes odor and taste). It could be that the concentration of OEO was a limiting factor in the acceptability of consumers (strong taste that makes it less desirable), because when it is added in lower concentration and mixture with copaiba essential oil (BEO), it had high acceptability. The same behavior was observed and reported by Ghabraie et al. 2016 [60] who found differences in the sensory evaluation of ground meat treated with combined Chinese cinnamon and essential oils of cinnamon bark, with the least acceptance of smell and flavor given to high concentrations of oils.

Regarding tenderness, it is possible that consumers detected a significantly higher shear force than presented in the CON treatment and reported a difference in the acceptability of beef patties from this treatment. Nascimento et al. 2020 [56], when evaluating edible coating containing cinnamon (*Cinnamomum zeylanicum*) and marjoram (*Origanum majorana* L.) essential oils of Wagyu hamburgers, which despite CON showing a significantly greater shear strength than all other samples, consumers did not notice these variations.

Overall acceptability usually is highly correlated with other sensorial attributes such as flavor or tenderness [61]. In the current study, overall acceptability was quite similar to flavor; the addition of exclusively oregano essential oil did not improve acceptability; however, the blend of both essential oils presented the highest overall acceptability.

### Microstructure

Similar characteristics were observed in previous studies with edible coatings containing essential oils of rosemary and oregano in the characteristics of beef, showing the droplets of essential oils throughout the meat [22]. In addition, they were similar to the structure found by Radünz et al. 2020 [62] for the essential oil of thyme encapsulated by spray drying (*Thymus vulgaris*) in the conservation of meat products similar to hamburgers. This verified the correct and homogeneous distribution of essential oils in the beef patties’ structure.

## Conclusions

The inclusion of oregano essential oil and the blend of oregano and copaiba essential oil increased antioxidant activity and decreased lipid oxidation compared to the control treatment (without the addition of essential oils), with higher antioxidant activity and lower lipid oxidation of these treatments for up to 21 days of display. Oregano essential oil increased chroma values and provided reduced values during exposure days, making the meat more attractive for a longer display period. The blend of essential oils (copaiba at 0.025% and oregano at 0.025%) in the hamburgers provided a lower cooking loss than the other treatments, with lower losses in the final display periods (days 14 and 21).

The mixture of essential oils also provided greater tenderness to the burgers compared to the control treatment, causing a progressive reduction in values from day 14 onwards, indicating softer meat after longer storage, in addition to improving the sensory attributes of the burgers. The essential oils studied increased the shelf life of the product, proving to be a potential option for the food industry to use natural ingredients instead of synthetic compounds to preserve the quality of processed foods. The combination of both essential oils (copaiba at 0.025% and oregano at 0.025%) would be a good alternative for this replacement of synthetic additives such as BHT because at low concentrations, they had a positive effect on shelf life in addition to increasing sensory properties evaluated by consumers.

## Supporting information

S1 File Ethics statement. (PDF)
