# Supplementary material for: Incorporation of copaiba and oregano essential oils on the shelf life of fresh ground beef patties under display: Evaluation of their impact on quality parameters and sensory attributes
Source: PLoS One. 2022 Aug 10;17(8):e0272852. doi: 10.1371/journal.pone.0272852 (PMC9365165; doi:10.1371/journal.pone.0272852)
Supplement: S1 File — (PDF) [file pone.0272852.s001.pdf]

PARECER CONSUBSTANCIADO DO CEP

DADOS DO PROJETO DE PESQUISA

Título da Pesquisa: Uso do óleo essencial de Copaíba e Orégano na elaboração de hambúrguer bovino gourmet: qualidade e aceitabilidade sensorial.

Pesquisador: jessica de oliveira monteschio

Área Temática:

Versão: 1

CAAE: 23282819.9.0000.5302

Instituição Proponente: Universidade Federal de Roraima - UFR

Patrocinador Principal: Financiamento Próprio

DADOS DO PARECER

Número do Parecer: 3.659.300

Apresentação do Projeto:

Trata-se de um projeto de pesquisa do curso de Zootecnia, da Universidade Federal de Roraima (UFRR). Introdução: Os extratos de óleos essenciais (OEs) são metabólitos secundários extraídos de várias partes de uma planta, incluindo folhas, flores, sementes, raízes e cascas (Benchaar et al.,2008). Segundo Hui (1996), os OEs apresentam compostos fenólicos capazes de interceptar e neutralizar os radicais livres responsáveis pelo processo oxidativo. De acordo com Souza et al. (2003), a indústria alimentícia visa à produção de alimentos inócuos e que apresentem vida longa de prateleira. A utilização de substâncias naturais de origem vegetal, torna o alimento mais atrativo ao consumidor por não apresentarem efeito tóxico, mesmo quando empregadas em concentrações relativamente elevadas. Além dos benefícios proporcionados à saúde,diversos estudos têm demonstrado o efeito inibidor de condimentos no desenvolvimento de microrganismos deterioradores e patogênicos veiculados por alimentos. (Pereira et al., 2006). A utilização de plantas aromáticas, possuidoras de óleos essenciais, geralmente com ações flavorizantes, comprovada ação antibacteriana e antioxidante, pode ser uma alternativa importante para a conservação de alimentos, diminuindo a concentração de aditivos sintéticos nesses produtos (Moreira et al.,2005).Tendo em vista a crescente preocupação dos consumidores com a segurança dos alimentos aliada a uma alimentação saudável, é notória a necessidade de encontrar novos compostos naturais com propriedade antimicrobiana. Atualmente o uso dos óleos essenciais como

aditivo em alimentos vem se difundido apresentado uma nova perspectiva de uso, com o objetivo de substituir alguns dos aditivos sintéticos, visando à conservação e consequentemente o aumento da vida de prateleira, além de proporcionar um sabor e aroma diferenciados (kempisk et al.,2017)O óleo essencial de copaíba tem sido estudado como alternativa por ser um produto natural, e um potencial manipulador da saúde humana. Os principais grupos de substâncias ativas de plantas existentes são: alcalóides, glucosídeos, compostos fenólicos, saponinas, mucilagens, flavonóides, taninos, óleos essenciais, etc (Martins et al. 2000).O óleo essencial de orégano possui alto conteúdo de compostos fenólicos, que têm sido considerados como os responsáveis pela sua atividade antimicrobiana (Souza et al., 2006). Os principais componentes antimicrobianos presentes no óleo essencial de orégano (OEO) são o carvacrol e o timol com comprovado efeito inibitório no controle in vitro da multiplicação de Salmonella (Nazer et al., 2005; Silva et al., 2005).Apesar das informações existentes sobre a utilização dos óleos essenciais na alimentação humana há necessidades de investigações mais profundas da ação dos princípios ativos e seus efeitos na alimentação, permitindo melhorar a qualidade dos produtos de origem animal e aumentando sua vida de prateleira, para que os óleos essenciais "naturais" possam ser adotados expressivamente alimentação humana. Justifica-se assim a realização do presente trabalho.

Hipótese: Os óleos essenciais irão melhorar os atributos de aceitabilidade (dureza, odor, sabor, suculência e aceitação geral), trazendo um novo aditivo ao mercado, de forma natural e trazendo benefícios a saúde humana.

Metodologia: As amostras de carne serão moídas e acrescidas de óleo essencial de Copaíba e Orégano em diferentes concentrações na mistura, preestabelecidos em outros trabalhos, mostrando o limite para aceitabilidade pelos consumidores e moldados. Serão seladas e armazenadas sob refrigeração a 4°C em condições equiparáveis às comerciais. A avaliação contará com aproximadamente 120 provadores não-treinados, sendo os participantes abordados e convidados a participar da análise sensorial. Professores, técnicos, funcionários e alunos do Centro de Ciências Agrárias localizada no campus Cauamé serão o público alvo da pesquisa, tendo uma maior amplitude da faixa etária entre os participantes (idade de 18 a 60 anos de idade). A avaliação será realizada em sala fechada, climatizada e ampla para evitar o contato entre os participantes e ocorrer influência das respostas entre eles. Cada sessão contará com 8 participantes por vez, que receberão as amostras de hambúrguer de cada tratamento (sem óleos essencial e com o óleo essencial de Copaíba e Orégano) e avaliarão em relação aos atributos de cor, sabor, odor, textura e aspectos globais. As amostras serão assadas até atingirem a temperatura interna de 71 graus e cortadas em pequenos cubos e apresentadas em recipientes

identificados com números aleatórios de três dígitos. Cada provador receberá as amostras e a ficha com o teste afetivo a fim de verificar a preferência e aceitação das amostras. O teste será composto de uma escala hedônica de 9 pontos, variando de desgostei muitíssimo (1) até gostei muitíssimo (9) (Breuer et al., 2000), além do termo de Consentimento Livre e Esclarecido e um copo de água e bolacha de água e sal para limpar o paladar entre a degustação de cada amostra. Será ainda realizada intenção de compra, com escala de três prontos sendo o 1 “certamente compraria”, o 2 “talvez comprasse” e o 3 “certamente não compraria”. Ao final da avaliação cada provador receberá um doce de forma a agradecer o tempo disponibilizado para a participação da análise sensorial que fará parte de um trabalho de pesquisa. Pelo método descritivo será feita a avaliação sensorial de hambúrgueres bovino de 5 tratamentos:CON (sem adição de óleo essencial); PAD (padrão, com antioxidante sintético (BHT) utilizado na industria alimentícia); Copaíba (com adição de 0,125% de óleo essencial de Copaíba); Orégano (com adição de 0,125% de óleo essencial de Orégano)e Blend Cop + Orég (com adição de 0,0625% de óleo essencial de Copaíba + 0,0625% de óleo essencial de Orégano) As amostras serão avaliadas por 120 provadores não treinados.
Critérios de inclusão: Para a pesquisa serão avaliados alunos maiores de 18 anos, técnicos e professores do Centro de Ciências Agrárias dos cursos de Zootecnia, Agronomia e medicina Veterinária, para se ter uma diversidade entre as idades e perfil dos consumidores.
Critérios de exclusão: Não abordará população indígena e população migrante.

Objetivo da Pesquisa:
Objetivo Primário: Objetiva-se avaliar os atributos sensoriais (dureza, odor, sabor, suculência e aceitação geral) de hambúrgueres d carne bovina adicionado de óleos essenciais.
Objetivos Secundários: Contribuir para as áreas de tecnologia dos produtos processados de origem animal, podendo os óleos essenciais atuarem como um antioxidante natural presente no alimento, proporcionando melhoras na qualidade do produto e aumentando sua vida de prateleira.

Avaliação dos Riscos e Benefícios:
Avaliação dos Riscos: Esta investigação pode oferecer desconforto e constrangimento ao sujeito, pois existe o risco de rejeição ao sabor do hambúrguer bem como risco de intoxicação alimentar em caso da amostra ofertada ser veiculadora de substâncias tóxicas, que podem ser, tanto químicas como de origem microbiana. O botulismo é um exemplo típico de intoxicação de origem bacteriana. Infecção está relacionada com a ingestão de carne e derivados contendo microrganismos com potencial infeccioso. Neste, as salmoneloses constituem típicos exemplos. Informamos que os riscos declarados acima são possíveis, e em caso de acometimento aos participantes declaramos a nossa inteira disponibilidade no auxílio médico e demais providências

que se façam necessárias ao tratamento de alguma possível enfermidade causada pela participação e promoção do total bem estar de alguma possível vítima. As amostras serão higienicamente armazenadas em geladeiras sob temperatura controlada e adequada para devida conservação e não contaminação por patógenos até o momento da análise. A manipulação no dia da análise será realizada por equipe devidamente treinada munida de itens que garantam a higiene no processamento como luvas, toucas e mascaras. As amostras serão submetidas a teste microbiológico para detecção de salmonella. O cozimento das amostras prevê ainda a eliminação de algum possível patógeno, mas de qualquer forma em caso de contaminação e danos saúde dos participantes será dada toda a assistência médica necessária conforme afirmação anterior.
Avaliação dos Benefícios: Com a análise sensorial os provadores terão oportunidade de aprender a distinguir através dos padrões de qualidade que o consumidor busca (dureza, odor, sabor, suculência e aceitação do produto final) além dos óleos essenciais agirem como um antioxidante que podem aumentar a vida de prateleira da carne e atuar na saúde humana como um antioxidante natural.

Comentários e Considerações sobre a Pesquisa:

Resumo: Estudos da utilização de antioxidantes naturais têm aumentado nos últimos anos, pois estes apresentam efeitos benéficos atuando na prevenção e redução de riscos a vários tipos de doenças que acometem a população. O presente trabalho se propõe a avaliar o uso do óleo essencial de Copaíba e Orégano nas principais propriedades da funcionalidade e da qualidade de hambúrguer durante períodos de armazenamento. Serão avaliadas propriedades de qualidade (perda por cocção, capacidade de retenção de água, cor (L*, a*, b*), maciez (Warner-Bratzler) e estabilidade oxidativa (TBARS, DPPH, FRAP e ABTS), relacionadas à funcionalidade e à aceitabilidade pelo consumidor. Devido à relevância comercial dos produtos de origem animal para o Brasil, destaca-se a importância da realização do presente trabalho tanto do ponto de vista científico quanto comercial e industrial.

Considerações sobre os Termos de apresentação obrigatória:

Todos os termos de apresentação foram apresentados.

Recomendações:

Recomenda-se inserir no TCLE, se houver, o nome dos pesquisadores envolvidos no estudo.

Conclusões ou Pendências e Lista de Inadequações:

Recomenda-se a aprovação do protocolo de pesquisa, pois não foram observados óbices éticos.

Considerações Finais a critério do CEP:

Este parecer foi elaborado baseado nos documentos abaixo relacionados:

Tipo Documento	Arquivo	Postagem	Autor	Situação
Informações Básicas do Projeto	PB_INFORMAÇÕES_BÁSICAS_DO_PROJETO_1449639.pdf	10/10/2019 09:18:40		Aceito
Outros	Ficha_de_analise_sensorial.pdf	10/10/2019 09:17:46	jessica de oliveira monteschio	Aceito
TCLE / Termos de Assentimento / Justificativa de Ausência	TCLE.pdf	10/10/2019 09:16:14	jessica de oliveira monteschio	Aceito
Projeto Detalhado / Brochura Investigador	Projeto.pdf	10/10/2019 09:16:05	jessica de oliveira monteschio	Aceito
Declaração de Instituição e Infraestrutura	autorizacao_de_pesquisa.pdf	10/10/2019 09:13:31	jessica de oliveira monteschio	Aceito
Folha de Rosto	Folha_de_rosto.pdf	10/10/2019 09:11:43	jessica de oliveira monteschio	Aceito

Situação do Parecer:
Aprovado

Necessita Apreciação da CONEP:
Não

BOA VISTA, 24 de Outubro de 2019

Assinado por:
Bianca Jorge Sequeira
(Coordenador(a))

Endereço: Av. Cap. Ene Garcez, nº 2413, UFRR, Campus Paricarana, Bloco PRPPG/UFRR, Sala CEP/UFRR.
Bairro: Aeroporto **CEP:** 69.310-000
UF: RR **Município:** BOA VISTA
Telefone: (95)3621-3112 **Fax:** (95)3621-3112 **E-mail:** coep@ufrr.br

FREE AND CLARIFIED CONSENT TERM

You are being invited to participate in the research "Incorporation of copaiba and oregano essential oils on the shelf life of fresh ground beef patties under display: Evaluation of their impact on quality parameters and sensory attributes" under the responsibility of the researcher: Jéssica de Oliveira Monteschio and your participation is not mandatory. At any time, you can withdraw from participation and you can leave the survey without prejudice to you or the researcher.

You are being invited to participate, as a volunteer, in a research on sensory analysis of gourmet beef burgers with different types of essential oils. This document will provide you with important information about the study. Please read the content below carefully and clarify your doubts with the team to decide whether or not you want to participate. If you accept to be part of the study, sign this document. If you do not want to participate you will not be penalized in any way.

The experiment aims to evaluate the sensory attributes (odor, tenderness, flavor, and overall acceptability) of gourmet beef hamburger added with Copaiba and Oregano essential oils as a "natural" additive. The samples offered will be in the form of cubes with a 2 cm edge size and will be subjected to a cooking process on a grill, preheated to 200 ºC, up to an internal temperature of 71 ° C.

The data will be collected at the Federal University of Roraima - UFRR, Campus Cauamé, and the samples will be produced in the Technology of Animal Products Lab of Animal Production course. For this, your participation is very important, and it would take place as follows: you will evaluate the samples from left to right with the option to repeat the evaluation of the samples already evaluated, if necessary. You must mark the numbers of the samples on the form, in the tables on the right and left, assign grades according to the scale presented on the form itself, which varies from 'dislike extremely' to 'like extremely'.

It is worth clarifying that this investigation can offer discomfort and embarrassment to the participant, as there is a risk of rejection of the hamburger flavor as well as a risk of food poisoning if the sample offered is a carrier of toxic substances, which can be both chemical and microbial in origin. Botulism is a typical example of intoxication of bacterial origin. Infection is related to eating meat and meat products containing microorganisms with an infectious potential. In this, salmonellosis are typical examples.

We inform you that the risks stated above are possible, and in case of involvement with the participants, we declare our entire availability in medical assistance and other measures that are necessary to treat any possible illness caused by the participation and promotion of the total well-being of any possible victim.

The samples will be hygienically stored in refrigerators under controlled temperature and suitable for proper conservation and non-contamination by pathogens until the moment of

analysis. The handling on the day of the analysis will be carried out by a properly trained team with items that guarantee hygiene in processing such as gloves, caps and masks. The samples will be subjected to a microbiological test to detect salmonella. The samples of cooking provides even eliminating any potential pathogen, but in any case in the event of contamination and damage health of the participants will be given all necessary medical care as earlier statement.

Regarding the benefits, it is worth noting that with the sensory analysis tasters have the opportunity to learn to distinguish through the quality standards that the consumer search (odor, tenderness, flavor, and overall acceptability) in addition to the essential oils act as a antioxidant that can increase the shelf life of meat and act on human health as a natural antioxidant.

It is noteworthy that your participation in this study is voluntary and spontaneous, without any type of remuneration during the period of the research, and you can give up at any time without prejudice to you.

An initial approach of the target group will be held in order to identify interest or not participation, which will be submitted to this term and orally prior to the project.

The information in this survey will be confidential and we guarantee that only the researcher will know about your participation.

You will receive a copy of this term with the telephone number and the institutional address of the main researcher and the CEP and you can answer your questions about the project and your participation, now or at any time. You can contact us, whenever you feel necessary, through the telephone number of the responsible researcher, Jéssica de Oliveira Monteschio, number (XX) XXXXX-XXXX, if you have any questions.

Jéssica O. Monteschio

Professor Jéssica de Oliveira Monteschio

I declare that I understand the objectives, risks and benefits of my participation in the research and agree to participate.

Research Participant
